# Environmental Distress Among Dutch Young Adults: Worried Minds or Indifferent Hearts?

**DOI:** 10.1007/s10393-025-01717-x

**Published:** 2025-05-27

**Authors:** Valesca S. M. Venhof, Bertus F. Jeronimus, Pim Martens

**Affiliations:** 1 System Earth Science, Maastricht University, Campus Venlo, P.O. Box 8, 5900 AA Venlo, The Netherlands; 2 Department of Environment and Health, GGD Groningen, Hanzeplein 120, 9713 GW Groningen, The Netherlands; 3 Faculty of Behavioural and Social Sciences, Developmental Psychology, Groningen University, Grote Kruisstraat 2/1, 9712 TS Groningen, The Netherlands

**Keywords:** evironmental change, psychological stress, mental health, youth, physical environment, solastalgia, environmental distress

## Abstract

Young adults increasingly report stress and concern over the erosion of their natural surroundings. We studied environmental distress and solastalgia in a representative sample of 1006 Dutch young adults aged 16–35 using an adapted version of the Environmental Distress Scale (EDS, Higginbotham ea., 2006). The role of ‘place attachment’, their sense of control, trust, and personality (BFI-10) was used to explore the psychological impact of environmental challenges close to home. Participants most often reported stress due to ‘noise’ (~ 22%), ‘disappearance of nature’ (~ 20%), and ‘heat’ (~ 18%). The latter two were described as most threatening. Environmental distress limited enjoyment of life in ~ 20% of youth and triggered worries about the future (~ 23%). A third of young adults reported worries that valued aspects in their home environment are being lost, with feelings of powerlessness (~ 27%) and limited trust in the Dutch government (~ 44%). A notable portion of surveyed young adults experienced environmental distress and solastalgia, but a substantial number appeared somewhat indifferent towards the state of their natural surroundings. To safeguard mental well-being and empowerment of the former group, as well as implement strategies to elevate environmental awareness and foster active engagement in the latter, more information on fundamental motivations and coping mechanisms is needed.

**Supplementary Information:**

The online version contains supplementary material available at 10.1007/s10393-025-01717-x.

## Introduction

‘The word he came up with was solastalgia, a portmanteau word of the Latin solus, which means ‘abandonment and loneliness’, and nostalgia’.

Humans often pride themselves in their plasticity and adaptability to various environmental changes and disasters (Bonnano et al., 2011; Evans, 1984; Potts, 1998). A mounting body of evidence and experiential insights (Furberg et al., 2011; Tollefson, 2022) underscore a disconcerting reality: ‘Earth is now well outside of the safe operating space for humanity’ (Richardson et al., 2023). Human impact through for example intensive agricultural practices, pollution, desertification, and the eutrophication of waterways via fertiliser use has reached staggering proportions (e.g. Almond et al., 2020; Watson et al., 2019). Globally, communities are confronted with their adaptive thresholds as rising sea levels and escalating natural disasters have become undeniable facts (e.g. Almond et al., 2020; Watson et al., 2019), driving an increase in chronic stress and mental illness (e.g. Clayton, 2021; Helbich, 2018a; WHO, 2022).

Many individuals, and especially adolescents and young adults, grapple with anticipatory threat due to environmental change that pushes humanity to the brink of its adaptive capacity (Léger-Goodes et al., 2022). Because youth are equipped with an extended time perspective, they tend to experience heightened awareness, concern, and emotional distress in response to the deteriorating condition of their home environment and the future of the planet (Gislason et al., 2021; Hickman et al., 2021; Lawrance et al., 2022). Concerns and threats about environmental change can trigger profound and prolonged states of environmental distress (Albrecht 2011; Cianconi, 2023), including conditions such as ‘climate anxiety’ (Clayton et al., 2020), ‘ecological grief’, ‘ecological anxiety’ (Ágoston et al., 2022; Comtesse et al., 2021; Cunsolo et al., 2020; Pihkala, 2022), and ‘solastalgia’ (Albrecht, 2005, 2007; Christensen et al., 2024; Galway et al., 2019; Leviston et al., 2023). The latter is coined by Glenn Albrecht (2005) to describe the emotional or existential distress caused by environmental change, particularly the loss of a beloved home environment.

Studies on the occurrence of environmental distress in young people become more common, yet most studies focus on older adult populations, most often threatened by environmental degradation due to intensive resource extraction (Canu et al., 2017; Cordial et al., 2012; Galway et al., 2019; Hendryx and Innes-Wimsatt, 2013). For example, a study on elderly Germans living in regions with open-pit coal mining showed the emergence of anxious, depressive, and somatoform symptoms (Krüger et al., 2022). Little is known about environmental distress in populations which are not directly exposed to acute environmental threats but report ‘chronic’ and cumulative distress from environmental stressors, as seen in densely populated and urbanised Western societies like the Netherlands. The Dutch National Institute for Public Health and the Environment (RIVM, 2023) reported how nearly half (~ 48%) of Dutch adolescent and young adults (aged 16–26) experienced (very) frequent stress. While the Dutch public became increasingly aware of the positive effects of a ‘healthy environment’ during the Covid-19 pandemic (Jeronimus, 2023), and recognises the value of living near green spaces and water (Van den Berg et al., 2015; De Vries et al., 2016), the potential role of the living environment on the levels of stress and overall mental well-being of individuals residing in the Netherlands remains understudied. In this paper, we examine the prevalence of environmental distress and solastalgia in a representative sample of Dutch young adults.

Stress is a complex and embodied concept that is often defined as involving situations where demands exceed one’s coping abilities (Cohen et al., 2013_,_ p.3), especially when these situations are unpredictable or uncontrollable and of high intensity (Epel et al., 2018). Physiological stress involves activation of the sympathetic-adrenal medullary system and the pituitary-adrenocortical axis (Baum et al., 1981), while psychological stress relates to cognitive interpretations or subjective perceptions of situations (Evans, 1984; Cohen et al., 2013). Environmental distress, through the lens of Lazarus ‘theory (Lazarus 1966; 1977, 1984), refers to how individuals assess stressors and evaluate their ability to cope with them (Cohen et al., 2013, p.5–6; Reser and Swim, 2011). This process involves primary appraisal (assessing harm, threat, or challenge) and secondary appraisal (evaluating coping resources and potential outcomes). Such simplified stress models artificially separate the somatic and mental stress responses (Epel et al., 2018), but can help grasp the impact of environmental changes like climate change. Solastalgia had already been observed to mediate the relationship between wildfire impact and mental health outcomes (Leviston et al., 2023).

**Figure 1:**
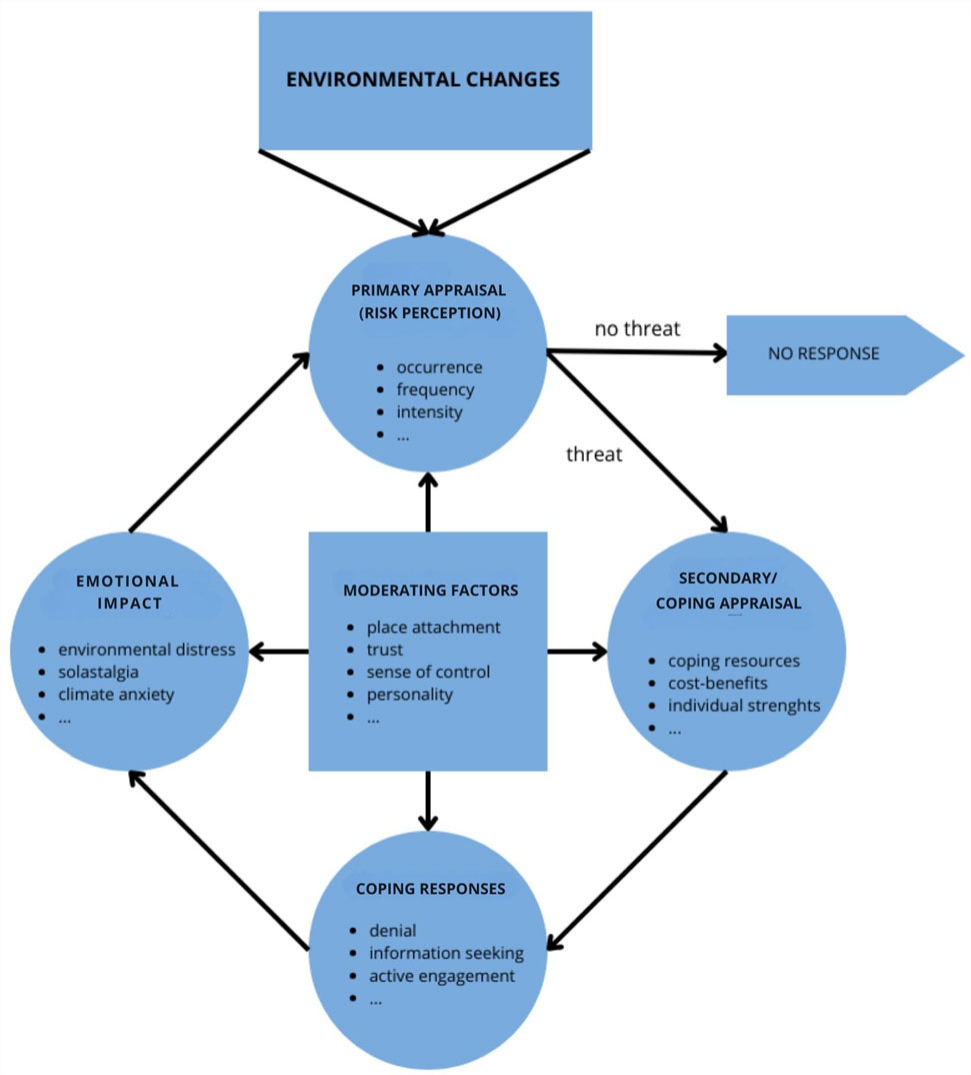
The environmental distress response. Model adapted from original model of Higginbotham (Higginbotham et al. 2006), their follow-up research (Higginbotham et al., 2014), the model of Lazarus (Lazarus 1966; Lazarus and Cohen 1977), and the model of Reser and Swim (Reser and Swim 2011).

This paper builds on Higginbotham’s framework on the ‘environmental distress response’ (Higginbotham et al., 2006) and their follow-up research (Higginbotham et al., 2014) in which they propose an ‘environmental cognitive stress model of global warming’, and is inspired by work of Lazarus (1966; 1977, 1984) and Reser and Swim (2011). The environmental distress response as such involves biopsychosocial factors influencing hazard perception, threat appraisal, perceived impact, and coping behaviours (Higginbotham et al., 2006). We investigated environmental distress, solastalgia, and whether factors such as attachment to place, sense of control, trust, and personality differences moderated the distress response (see Fig. 1). Exploring environmental distress and solastalgia in Dutch young adults can provide valuable insights into the complex interplay of factors influencing youth mental well-being and the need for preventive measures. Furthermore, this knowledge can inform collective and government policy decisions to mitigate and adapt to environmental stressors (Willox et al., 2013), and help to empower young people to engage in sustainable behaviours (Brosch and Steg, 2021), nature conservation, and pro-environmental activism (Ojala, 2013).

## Materials and Methods

### Study Design and Sampling

This questionnaire-based study included 1006 young adults aged 16–35 living in the Netherlands, a country with ~ 18 million inhabitants spread across 12 regional provinces. The Western parts of the country are most densely populated (e.g. South Holland with ~ 1391 inhabitants per km^2^). According to Dutch government statistics, 28% of the Dutch population was younger than 25 years in 2022.

Questionnaires were provided to a nationally representative sample (stratified by gender, age, education level, and province of residence) and reached out by Flycatcher Internet Research (www.flycatcher.eu). This company has a quality mark for market, opinion, and social research (ISO 20252), and an environmental standard (ISO 14001). Representativeness was judged using the so-called ‘golden standard’ 1 calibration instrument, specially developed by the Data & Insights Network (formerly MOA), in collaboration with Statistics Netherlands (CBS 2022).^2^ Panel members were invited by e-mail to participate in the survey. They used a personal hyperlink to the digital questionnaires. Participants were eligible if they were between 16 and 35 years old, lived in the Netherlands, could read/write in Dutch, and provided informed consent.

### Measurement Instruments

The study had three sets of questionnaires. The first set assessed thirteen demographically oriented items and captured scores on the big five personality factors (extraversion, agreeableness, conscientiousness, emotional stability, and openness), using the Dutch version of the 10-item BFI-10 (Rammstedt, 2007). Additionally, participant’s self-perceptions regarding their (mental) health were gauged with slider bars (range 1–100) on which they scored their happiness, depression, anxiety, loneliness, stress, general health (both current and anticipated in ten years), and their perspective on the well-being of their home environment (both present and expected in ten years).

The second part of the questionnaire measured environmental distress using subcomponents derived from or inspired by the Environmental Distress Scale (EDS); see Supplementary file 1, Table S1 for psychometric details. The original EDS components were identified by Connor et al. (2004) in their qualitative research in the Upper Hunter Valley of Australia, where open cut mining caused invasive environmental changes (Connor et al., 2004). The original EDS was validated by Nick Higginbotham and colleagues (2006) by comparing two communities in the Upper Hunter Region of New South Wales, one with and one without exposure to open-pit coal mining. The EDS has been successfully administered near two open-pit mining areas in Western Germany (Krüger et al., 2022). Furthermore, the EDS-Solastalgia has been applied in drought-affected areas of New South Wales in Australia (Sartore et al., 2008), after wildfires in Arizona in the USA (Eisenman et al., 2015) and Australia (e.g. Christensen et al., 2024; Leviston et al., 2023; Stanley et al., 2024).

Our Dutch version of the EDS included four original EDS components (Higginbotham et al., 2006), of which the dimensions ‘frequency’, ‘threat’, and ‘felt impact’ were translated into Dutch and adjusted for the Dutch context. The fourth, ‘solastalgia’, was directly translated into Dutch. This 9-item ‘solastalgia scale’ was adopted from Higginbotham’s 2007 reworded version written for the Hunter Community/Cohort Study to be generic across local environments where people live. The translation of the components of the questionnaire followed the guidelines by Tsang and colleagues (2017) and the Dutch guidelines for writing accessible Dutch (‘plain’ or B1 level, see Accessibility, 2023).

The third part of the questionnaire examined climate change and related emotions and was not part of this paper. After online programming, all three questionnaires were piloted internally by two researchers working at Flycatcher (other than the responsible researcher), and externally by three volunteers. The original Dutch version of the first and second part of the survey can be found in Supplementary file 2, with an English translation in Supplementary file 3. The survey was eventually launched online on Monday 27 February and completed on Thursday 9 March 2023.

#### Moderating Factors

We explored four moderating factors, defined as factors influencing the strength or direction of the perceived level of environmental distress, which were ‘place attachment’ (original EDS version, translated), a self-designed 4 item component on ‘sense of control’, ‘trust’ (adjusted from original EDS and translated), and ‘personality’ (Dutch version of BFI-10). In the original model of Higginbotham et al. (Higginbotham et al. 2006), a ‘sense of place’ was thought to influence the environmental distress response at the level of ‘threat appraisal’ and experienced impact. This emotional bond to one’s living environment, or ‘place attachment’, shapes our sense of belonging and identity (Albrecht, 2011; also, Galway et al., 2019; Morgan, 2010.). Place attachment drives individuals to preserve cherished landscapes and mourn change and intensifies environmental distress (Scannell and Gifford, 2010; Altman and Low, 2012; Maller et al., 2008). Yet, green places offer solace (Higginbotham et al., 2006). Place attachment influences risk perception and coping after disasters (Bonaiuto et al., 2016) and fosters mental resilience and environmental stewardship (Willox et al., 2012; Conrad, 2017; García-Martín et al., 2018).

The environmental distress response is also shaped by one’s perceived control over stress (Cohen et al., 2013); a higher level of perceived control mitigates the impact of events and reduces mental health issues (Cohen et al., 2013, p.47; Galway et al., 2019; Hovenkamp-Hermelink et al., 2019). Furthermore, personality traits and government trust among others impact individuals’ response to environmental stressors (Hickman et al., 2021; Larsen et al., 2025; Ojala, 2013; Reser and Swim, 2011).

#### Ethical Considerations

All respondents provided written informed consent before starting the survey. Respondents received a small contribution for participation in the study in terms of points from Flycatcher and their partner agency (with a value of three euros for this survey) which they can exchange for a gift voucher or a donation.^3^ As the questionnaire focuses on mental health, respondents were given information about where to seek psychological support if needed.

### Data Analysis

The online survey was accessible for participants until > 1000 questionnaires were completed by a sample that exhibited reasonable national representativeness of Dutch youth. An independent researcher of Flycatcher examined the data and excluded incomplete questionnaires from further analysis. Descriptive statistics were calculated using IBM SPSS Statistics (version 28.0.1.0) and compared with data from the ‘golden standard’ (see study design) to confirm the ‘representativeness’ of the sample for the Dutch population aged 16 to 35 years, with respect to gender, age, education level, and province of residence. Second, we created scale scores and derives frequencies for personality, place attachment, solastalgia, perception of environmental stressors, threat to self or family, felt impact, sense of control, and trust. Results were visualised in bar charts. Cronbach alpha scores for the individual component scales are provided in Supplementary file 1, Table S1. We analysed the data only when a participant had completed the questionnaires in full, ensuring there were no missing items, as all Likert scales had to be answered before participants could submit and save the questionnaire.

The ‘solastalgia’ and ‘place attachment’ scale scores and their mean and standard deviation (SD) were calculated, as well as scales for five personality factors (BFI-10, Rammstedt, 2007). We applied Spearman correlations (r) for individual Likert scale data and sum scores. Correlations were typically classified as small in effect magnitude between 0.10 and 0.19, moderate between 0.20 and 0.29, and large from 0.30, based on effect sizes commonly found in social psychology (Richard et al., 2003). We restrict our discussion to correlations with an effect size of *r* = 0.20 or larger; following the threshold commonly observed in studies in personality and social psychology over the past century (Richard et al., 2003), as such effects are more likely to be meaningful and replicable. For this, studies need up to 250 participants to reduce estimation error in correlations (Schönbrodt & Perugini, 2013) which our study sample met easily. Given the explorative nature and design of our study, we accentuate practical significance (effect sizes) and not statistical significance (*p*-values), which means we adhere to conventional *p*-values unadjusted for multiple testing (Cohen, 1990; Nakagawa, 2004).

## Results

### Survey Sample

Of the 1261 respondents who started the online questionnaire, 32 provided no informed consent or were not living in the Netherlands, and 159 did not complete the questionnaire for unknown reasons. Flycatcher removed another 64 of the remaining 1102 who completed the questionnaires because of poor response quality (5.8%); open answers, inconsistency of answers, straight lining (same answer to a series of items), and unrealistic completion time. The final dataset comprised 1006 respondents. A flowchart is provided in Supplementary file 4 as Figure S1.

### Sociodemographics

Our sample was representative for the Dutch population in terms of age, gender distribution, education level, and province of residence, as outlined in Table 1. The sample was on average 25.9 years old (SD = 5.6). There was a slight overrepresentation of women and higher educated participants (see Table 1) and some variability in the proportion of participants from the specific provinces (Drenthe/Friesland slightly overrepresented and North-Brabant/North-Holland slightly underrepresented). The distribution urban versus rural was in line with the golden standard (CBS). Participants typically lived 2.65 years (SD = 1.04) 4 in their home environment and many (~ 40%) lived there up to five years, or even longer than a decade (~ 30%). Finally, ~ 14% of the respondents reported to have a profession related to nature or climate, and ~ 13% were active in an environmental organisation.

**Table 1:** Sociodemographics.

Characteristics		Study sample		CBS 2022^ii^	**▲**
		*N*			
		1006	100%		
Age	16–20	243	24.2%	24%	
	21–25	243	24.2%	25%	
	26–30	260	24.2%	26%	
	31–35	260	24.2%	25%	
Gender	Women	515	51.2%	49%	
	Men	489	48.6%	51%	
	Other	2	0.2%		
Education	Practical	241	24%	24%	
	Middle	526	52.3%	46%	●
	Theoretical	239	23.8%	40%	●
Marital status	Single		39.6%		
	Married		14.1%		
Residency	Urban	618	61.4%		
	Rural	388	38.6%		
	House owner		57.9%		
	With children		42.6%		
Provinces*	Drenthe	46	4.6%	2%	●
	Flevoland	25	2.5%	3%	
	Friesland	60	6.0%	3%	●
	Gelderland	114	11.3%	12%	
	Groningen	42	4.2%	4%	
	Limburg	65	6.5%	6%	
	Noord-Brabant	122	12.1%	14%	●
	Noord-Holland	144	14.3%	18%	●
	Overijssel	59	5.9%	7%	
	Utrecht	80	8.0%	8%	
	Zeeland	24	2.4%	2%	
	Zuid-Holland	214	21.3%	22%	
Duration of living in home area	< 1 yr	127	12.6%		
	1–5 yrs	400	39.8%		
	5–10 yrs	174	17.3%		
	> 10 yrs	305	30.3%		
Profession	Yes	116	11.5%		
Nature/climate**	No	741	73.7%		
Active in environmental	Yes	130	12.9%		
organisation	No	876	87.1%		

*missing 11 (1.1%)** missing 149 (14.8%) ▲ = significant differences between proportion of the brackets in the study sample from comparable brackets in the Dutch CBS population data.

^ii^https://www.cbs.nl/nl-nl/visualisaties/dashboard-bevolking

On average (scale 0–100), the 1006 young adults indicated that they were happy (Mean = 63.4 (SD = 20.5), but also somewhat lonely (Mean = 31.9, SD = 26.3), anxious (Mean = 32.3, SD = 25.7), depressed (Mean = 38.4, SD = 23.7), and especially stressed (Mean = 43.4, SD = 25.1). Furthermore, they rated (scale 0–100) their general health fairly positive (Mean = 68.8, SD = 21.6), and the health of their environment comparably (Mean = 66.9, SD = 19.0). Finally, participants expected that their own health would improve slightly over the next decade (to Mean = 70.3, SD = 19.6) while the health of their environment was expected to decline (to Mean = 65.9, SD = 19.8).

#### Environmental Stressors and Threat Appraisal

**Figure 2:**
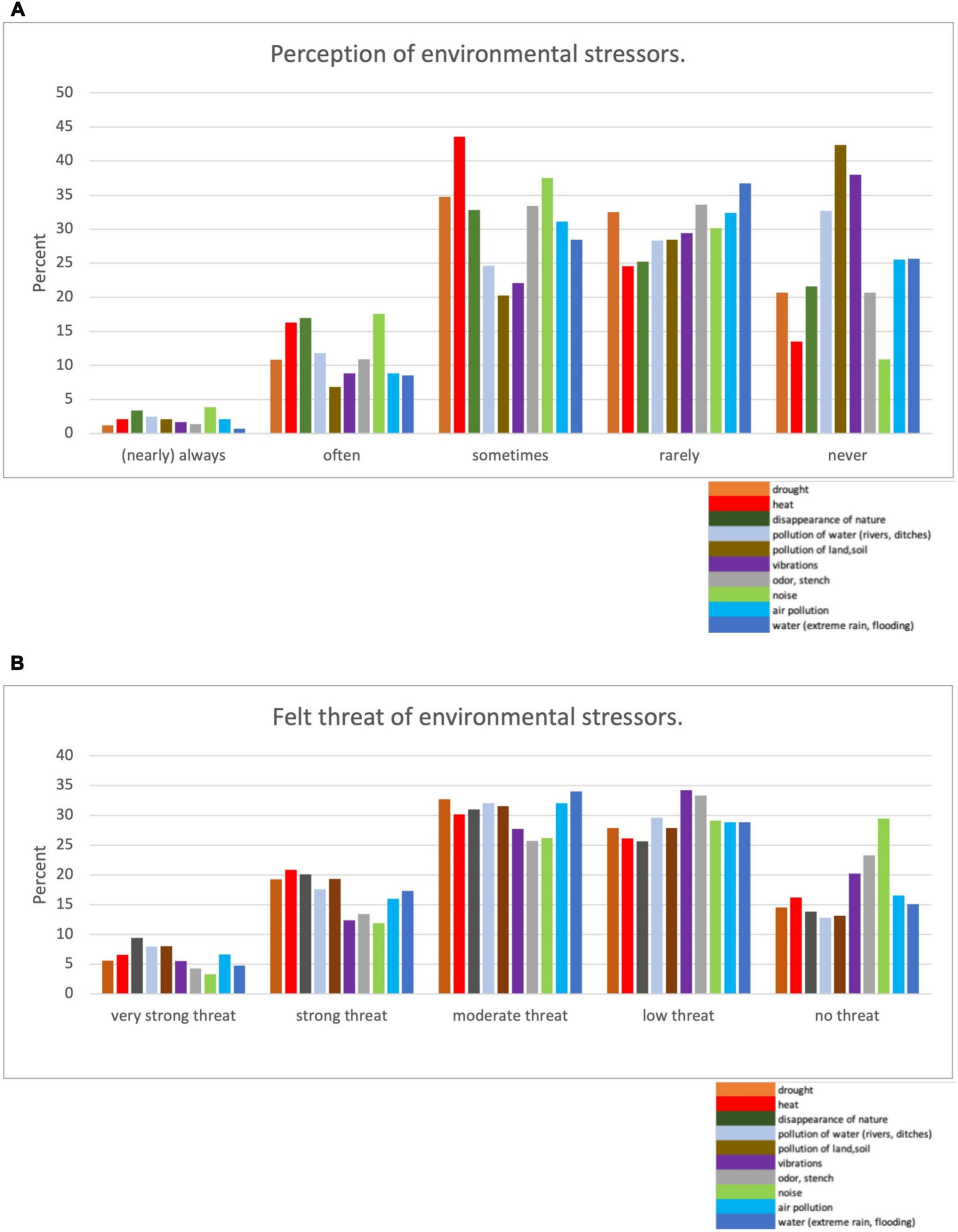
Perception (**A**) and felt threat (**B**) of environmental stressors.

A densely populated country such as the Netherlands can present young adults with many environmental stressors and most (~ 97%) respondents encountered at least one environmental stressor in their home environment. The reported frequencies of occurrence of nine different stressors in the home environment are presented in Fig. 2A. The respondents experienced most stressors ‘rarely’ or ‘never’, but the top three of stressors that were reported most frequently were ‘noise’ (~ 22%), ‘disappearance of nature’ (~ 20%), and ‘heat’ (~ 18%). All stressors, except ‘disappearance of nature’ and ‘drought’, were more frequently experienced (*p* ≤ 0.05) by young adults living in urban versus rural area. Females experienced ‘noise’ (*d* = 0.24 (SE = 0.06), *t*_(1002)=_3.81, *p* < 0.001) and ‘disappearance of nature’ (*d* = 0.21 (SE = 0.07), *t*_(1002)_ = 0.81, *p* = 0.002) more frequently compared to men. All stressors were more frequently (*p* < 0.001) experienced by people that were active in an environmental organisation, compared to those who were not. This was also found for people having a profession related to nature or climate (*p* ≤ 0.05), except for ‘noise’.

Of the 955 respondents who identified at least one stressor as a threat to health, the majority reported a low-to-moderate level of threat. The top perceived threats included ‘disappearance of nature’ (~ 30%) and ‘heat’ (~ 28%), as illustrated in Fig. 2B. Except for ‘heat’ and ‘drought’, stressors were rated significantly higher in terms of threat level (*p* ≤ 0.05) by urban versus rural youth. Furthermore, those actively engaged in environmental organisations of professions related to nature or climate reported significantly higher levels of threat (p < 0.001) for all stressors except for pollution of the soil.

### Felt impact of Environmental Stressors: Mind and Body

The felt impact (~ 97%) on their psychological, cognitive, and social daily functioning (Table 2) was relatively high (median = 71 on range 25–96), with mean impact of 53.5 (SD = 11.9), and an approximately normal distribution (skewness = 0.056, SE = 0.084). Many participants reported that the perceived environmental stressor(s) resulted in a diminished ability to enjoy life (~ 20%), feelings of depression (~ 19%), and a ‘loss of concentration’ (~ 19%) that impacted on their daily lives. They worried about the future (~ 23%) due to the identified stressors in the home environment, which could (~ 14%) or did (~ 11%) cause them or their family health problems and were thought to reduce economic value of their home (~ 15%). Felt impact was significantly higher for those having a profession related to nature or climate, compared to those who did not (*d* = 5.70 (SE = 1.24), *t*_(719)_ = 0.28, *p* < 0.001); and for those being active in an environmental organisation, compared to those who were not (*d* = 5.72 (SE = 1.18), *t*_(846)_ = 0.22, *p* < 0.001).

**Table 2:** The felt impact of environmental stressors.

Due to the environmental problems in my home environment:		N	Proportion (totally) agree %
1	I’m less able to enjoy life	197	19.6
2	I’m experiencing joy	164	16.3
3	I become depressed	186	18.5
4	I’m angry	175	17.4
5	I’m anxious	159	15.8
6	I experience tension, stress	173	17.2
7	I’m less able to concentrate	191	19.0
8	I worry a lot	164	16.3
9	I cannot sleep well	169	16.8
10	I’m less able to perform in study and/or work	150*	14.9
11	I’m less able to function well at home	149	14.8
12	I feel an improvement in my daily functioning	90	9.0
13	I experience health problems	107	10.6
14	I feel hindered in hobby/sports/social activities	142	14.1
15	I think people in my home environment are getting ill	136*	13.5
16	I think I personally can become ill	143	14.2
17	Is my house worth less	148*	14.7
18	There are tensions and/or arguments in my neighbourhood	129	12.8
19	I have concerns about the future	230	22.9
20	It will only become better to live in my neighbourhood in the coming years	130	12.9

*N* = 977.

*these questions included an option ‘N/A’. Here, total respondents (N) were 930, 922, and 898 respectively.

### Solastalgia

Table 3 presents the prevalence of solastalgia, with a mean score of 24.6 (SD 6.5, median 25.0, range 9–45), demonstrating an approximately normal distribution (skewness 0.070, SE = 0.084). In Higginbotham’s original study (2006), two towns in the Upper Hunter region were contrasted: one in a ‘low impacted’ environment and another in a ‘high impacted’ (by mining) area. Solastalgia mean scores of these areas were 20.8 (SD 6.3) and 28.3 (SD 7.2) respectively (Higginbotham et al., 2006). In comparison with our sample of young Dutch adults, acknowledging contextual differences, our solastalgia scores fall between the scores in Higginbotham’s low and high impacted areas. In our sample, ~ 36% of the respondents was (very) worried that valued aspects of their area were lost (Item 3) and ~ 27% of them felt (very) powerless to stop unwanted changes in their living environment (Item 9, see Table 3 for details). No differences in solastalgia were observed between gender or those who lived in rural versus urban areas. Solastalgia scores were significantly higher for those having a profession related to nature or climate, compared to those who did not (*d* = 4.02 (SE = 0.67), *t*_(715)_ = 0_._83, *p* < 0.001); and for those being active in an environmental organisation, compared to those we were not (*d* = 4.43 (SE = 0.63), *t*_(839)_ = 0.06, *p* < 0.001).

**Table 3:** Dutch EDS, results.

			Strongly agree	Agree	Neither Agree nor disagree	Disagree	Strongly disagree	N/A
			%	%	%	%	%	%
*Solastalgia (N* = *841)*								
	1	My sense of belonging to this place has been undermined by unwelcome change	2.2	20.5	29.9	35.2	7.7	4.6
	2	I’m sad that familiar aspects of this place are disappearing (e.g. animals, plants, landmarks, open space)	3.7	23.4	28.1	30.3	7.7	6.9
	3	I’m worried that aspects of this area that I value are being lost	3.7	32.3	26.0	27.3	6.8	3.9
	4	I miss having the sense of peace and quiet that I once enjoyed by being in this place	3.3	16.8	30.0	33.7	9.7	6.5
	5	I am upset about the way this area looks now	2.1	12.8	25.2	42.5	13.4	3.9
	6	My lifestyle is being threatened by environmental change in my local area	2.1	12.1	27.4	39.1	15.2	4.1
	7	Unique aspects of nature that made this place special are being lost forever	3.1	15.7	29.4	34.7	11.6	5.5
	8	I’m saddened by unwelcome change I see in my landscape	2.8	19.9	26.8	35.7	10.1	4.7
	9	I feel powerless to stop unwanted changes to this place	5.2	22.1	28.9	30.6	8.4	4.8
*Place Attachment (N* = *1006)*								
	1	I’m proud of the heritage of this place	5.2	39.6	41.6	12.9	0.8	
	2	I would continue to live in this place even if I were given the opportunity to leave	8.7	34.1	27.3	25.0	4.9	
	3	My sense of who I am is linked to the environment where I live	4.3	35.6	29.8	25.1	5.2	
	4	I get comfort or peace of mind from this place	7.0	43.8	30.8	15.0	3.4	
	5	I feel I know every rock, nook, and cranny around these parts	8.8	36.9	26.5	23.3	4.5	
	6	I feel a deep connection to this place	6.0	28.1	32.6	27.2	6.1	
	7	I feel a sense of responsibility to the people of this place	2.8	24.7	39.3	25.0	8.3	
	8	I feel I have a duty to maintain the land for future generations	6.1	32.1	36.8	19.9	5.2	
	9	Because of the changes to this place, I would leave I could	4.2	18.5	36.3	34.7	6.4	
Sense of control (*N* = 1006)								
	1	I can do something myself to make my home environment healthier	3.2	33.2	41.8	20.0	1.8	
	2	My way of living causes damage to my home environment	1.6	10.2	27.9	47.9	12.3	
	3	I have influence on decisions regarding my living environment	2.2	23.5	35.9	31.9	6.6	
	4	I’m actively involved in protecting and improving (health of) my home environment	4.7	31.2	37.2	22.9	4.1	
Personality			Mean	SD	Range	skewness		
	Extraversion (*N* = 793)		7.4	1.6	2–10	−.645 (SE .087)		
	Agreeableness (*N* = 765)		7.3	1.5	3–10	−.803 (SE .088)		
	Conscientiousness (*N* = 791)		7.7	1.4	2–10	−.778 (SE .087)		
	Emotional stability (*N* = 777)		5.7	2.0	2–10	.292 (SE .088)		
	Openness (*N* = 742)		6.6	1.9	2–10	−.178 (.090)		

As shown in Table 4, all four Environmental Distress Scale dimensions of stressor frequency, threat, felt impact, and solastalgia were strongly and significantly correlated. For the moderating factors, sense of control had a (moderately) strong correlation with the four EDS dimensions, and with place attachment. Furthermore, the personality trait ‘emotional stability/neuroticism’ was correlated with ‘frequency’ of environmental stressors and felt impact (see Table 4). A full overview of all correlations, range of N’s, and p-values can be found in Supplementary file 5, Table S2.

**Table 4:** Correlations.

	1	2	3	4	5	6	7
1. Frequency		**.82**	**.48**	**.45**	−.04	**.20**	**.21**
2. Threat	**.82**		**.45**	**.39**	−.00	**.25**	.18
3. Felt impact	**.48**	**.45**		**.52**	−.05	**.26**	**.25**
4. Solastalgia	**.45**	**.39**	**.52**		.08	.**25**	.19
5. Place attachment	−.04	−.00	−.05	.08		.**27**	−.14
6. Sense of control	**.20**	**.25**	**.26**	**.25**	**.27**		−.03
7. Emotional stability	**.21**	.18	**.25**	.19	−.14	− .03	

All correlations presented here bold were at least of moderate effect magnitude and significant at *p * < .001. A full overview of all correlations, range of N’s, and all *p * = values can be found in Supplementary file 5, Table S2.

### Place Attachment

The place attachment scores for our 1006 participants were surprisingly high (Mean = 28.4 (SD = 5.4), median 29.0, range 11–45, skewness − 0.199 (SE.077)), which is above the place attachment score as measured in Higginbotham’s low (Mean = 16.5 (SD = 5.6) and high (Mean = 20.8 (6.3)) impacted area (Higginbotham et al. 2006, Table 3). Dutch young adults living in rural area reported significantly higher place attachment (*d* = 1.57 (SE = 0.38) compared to those who lived in urban area (*t*_(1004)_ = 4.14, *p* < 0.001). Males had a significant higher place attachment (*d* = 1.29 (SE 0.37), *t*_(1002)_ = 0.11, *p* < 0.001) compared to females.

Dutch young adults were generally proud of their local heritage (~ 45% (strongly) agreed and ~ 14% (strongly) disagreed), with half of them feeling comfort or peace of mind from being in their home environment (~ 51%/ ~ 18%), which they seemed to know quite well (‘I know every rock nook, and cranny’, ~ 46%/ ~ 28%). The respondents were indifferent about whether they felt a deep connection to their place (~ 34%/ ~ 33%) or whether their sense of place related to their identity (34%/33%). Most Dutch youth did not feel a strong sense of responsibility for others in their home environment (~ 28% (strongly) agreed while 33% (strongly) disagreed), or duty to maintain the land for future generations (~ 38%/ ~ 25%, respectively). A large minority (~ 43%) would continue to live in their home environment even when they were able to leave (and ~ 41% would not live elsewhere), but almost up to a third would leave (~ 30%) when given the opportunity.

### Sense of Control

Many participants (36.4%) (totally) agreed that they could personally help to improve the health of their living environment, and only 21.8% (totally) disagreed. A third (35.9%) of youth was already involved in protecting and improving their living environment, although many (~ 39%) felt they lacked all influence. Only ~ 26% experienced some control over decisions related to the health of their living environment. Most (~ 60%) respondents reported that their own behaviour and way of living were (totally) not damaging their living environment. One-fifth (~ 19%) of respondents rated economy and employment opportunities (jobs) as more important than a healthy living environment, but two-fifth (~ 41%) (totally) disagreed with this statement. The mean score on ‘sense of control’ for 1006 participants was 11.5 (SD 2.5, range 4–20, skewness 0.001 (SE 0.077)). Young adults with a profession related to nature or climate reported more control than those with other jobs (*d* = 1.10 (SE = 0.25), *t*_(855)_ = 0.52, *p* < 0.001). This was also true for youth active in an environmental organisation, versus those who were not (*d* = 1.59 (SE = 0.23), *t*_(1004)_ = 0.33, *p* < 0.001).

### Trust

Dutch young adults often (~ 44%) had little to no trust in the Dutch government’s capacity to tackle environmental problems including climate change, which is double the proportion (~ 22%) of youth who had (very much) trust in their government. Trust in European policy makers such as in the European Union was slightly larger but a third (~ 33%) still reported little to no trust and a comparable group (~ 28%) reported (strong) trust. Participants were even more indifferent about their own municipality, as one quarter reported (strong) trust (~ 25%) or no or little trust (~ 27%) that they would be able to handle environmental change. Youth described more trust in international organisations such as WHO and IPCC (~ 40%/ ~ 24%), and Dutch climate organisations (~ 38%/ ~ 25%). Half of the respondents had no or little trust in industry, and only a minority did trust them (~ 19%). On the other hand, trust in the medical sector was quite high, with (very much) trust in medical doctors and nurses (~ 44%) and only few (~ 17%) had no or little trust, also in general practitioners (~ 40%/ ~ 21%), and the Dutch public health department (~ 35%/ ~ 25%).

### Personality

The mean scores for the five BFI-10 personality traits are provided in Table 3. A higher score on emotional stability/neuroticism was correlated (*p* < 0.001) with a higher experienced frequency of environmental stressors and a higher distress impact score (see Table 4).

## Discussion

Young adults face numerous challenges, and their mental well-being is shaped by a multitude of factors. In this intricate web shaping emotional wellness, the psychological stress due to the state of the natural living environment receives only marginal attention. This paper enriches the literature with a first estimate of environmental distress and solastalgia in a representative sample of 1006 Dutch young adults (aged 16–35), also called the ‘generation of hopes and dreams’ (Levinson, 1986).

Our study results yielded five key observations. First, environmental noise, heat (~ 28%), and the disappearance of nature (~ 30%) were the most frequently reported stressors in daily life, and the latter two were judged as most threatening to (mental) health. Second, ~ 20% of young adults reported daily life disruption due to environmental stress and ~ 23% worried about their future health in relation to the state of the home environment. Third, participants exhibited a significant degree of ‘emotional indifference’, lacking emotional involvement, concern, or interest, towards both their environment and their personal responsibility, with approximately 60% perceiving their actions as inconsequential to environmental matters. Fourth, one quarter of Dutch young adults experienced powerlessness (~ 27%) and many had low trust that various government bodies were able to handle environmental degradation. Finally, we present an adapted version of the Environmental Distress Scale (EDS; Higginbotham et al. 2006) in which the solastalgia subscale was applied to the Western local context, which proved to be a fruitful approach. These key observations are now discussed in more detail below.

### Noise

Although ‘noise’ ranked among the top three stressors in the home environment in terms of frequency, our participants considered it the least threatening to health and well-being (~ 15%) compared to the nine other stressors (see Fig. 2B). Recent research by the Dutch Public Health Agency (van Pol & Simon, 2023) also found that noise, particularly from nearby sources such as ‘road traffic, neighbours, and construction activities’, had a significant impact on Dutch residents. Despite various efforts to increase public awareness about the effects of noise pollution on well-being (WHO, 2018), our research might indicate a lack of recognition of this correlation among the Dutch population.

### Solastalgia

In the original Environmental Distress Scale (EDS), the solastalgia subscale was applied to residents living in a farming region damaged by large-scale open-pit coal mining. We translated and adapted the questionnaire to be employed to measure common local environmental problems in general Dutch neighbourhoods. Our Dutch version demonstrates high applicability and strong intercorrelations across the four dimensions (B-E, see Supplementary file 1, Table S1), even when applied at a broader population level, as exemplified in the Netherlands. The solastalgia mean score of the surveyed population was considerable (M = 24.6, SD = 6.5) and one-third of young adults worried that valued aspects of their natural habitat were being lost. Such ‘anticipatory’ environmental worries and stress in young people have been observed in other countries (Hickman et al., 2021). The young adults who perceived themselves as powerless also reported low trust in government bodies to address environmental issues, including climate change, which may increase their risk of stress and mental health problems (Cohen et al., 2013, p.47; Galway et al., 2019; Hovenkamp-Hermelink et al., 2019).

Environmental concerns can prompt environmental action and engagement; however, they can serve as positive and active coping mechanisms (Stevenson and Peterson 2015). Our study was not designed to investigate the relationship between environmental concern and environmental action, but we did observe that both the frequency, threat, and impact of environmental stressors, and level of experienced solastalgia was higher in young adults being environmentally active. Empowering young people through education, involvement in decision-making processes, and fostering resilience can enhance their sense of control (Larsen et al., 2025). Providing tools for problem-solving, encouraging ‘critical thinking’, and promoting environmental stewardship can help them feel more capable and engaged in addressing environmental challenges. Our preliminary results align with previous work that show the complex relationships between environmental concerns, stress, empowerment, and environmental action (Reser, 2011; Steg, 2023). The many young adults who felt powerless (~ 27%) suggest that the choice to leave the powerless items out of the brief Solastalgia scale with 5 items (Christensen et al., 2024)) may prove an important omission.

#### Shifting Baseline Syndrome

One of the most worrisome observations was the significant ‘emotional indifference’ exhibited by a substantial portion of our sample towards their natural surroundings. Two potential rationales underpin these findings, revolving around ‘shifting baselines’ and the pervasive impact of growing up in environments lacking in nature. The human perception and assessment of (impending) environmental hazards are multifaceted, influenced by a myriad of factors. ‘Western’ generations, exemplified by our Dutch sample, are imbued with diverse expectations and encounters regarding their relationship with the natural world. A predominant emphasis on industrialisation, urbanisation, and economic expansion has gradually altered our foundational perceptions of the environment, fostering a heightened tolerance towards progressive ecological deterioration (Soga and Gaston, 2018). This phenomenon, termed ‘Shifting Baselines Syndrome’, was initially elucidated by marine biologist Daniel Pauly in 1995, primarily within the context of overfishing. Pauly contended that we have become disconnected from nature, disregarding historical shifts and misconstruing the present state as natural (Bolster et al., 2012). Moreover, Dutch children are increasingly growing up in an environment characterised by a ‘nature deficit’ (Louv, 2008). This deficiency translated to diminished outdoor engagement, resulting in a depletion of ecological knowledge (Pilgrim et al. 2008), and a detachment from their natural surroundings.

On the other hand, a significant minority of our respondents reported worries about the disappearance of nature. They showed strong attachment to their natural habitat, scoring above averages found in Australian towns (Higginbotham et al., 2006). Dutch young adults were generally proud of their local heritage (~ 45%) and half of them felt comfort in their home environment. Although not statistically tested, the original EDS proposed that a stronger sense of place would intensify the level of experienced threat from environmental change, including solastalgia (Higginbotham 2006). Other studies describe that strong place attachment can drive proactive preservation efforts (Schultz, 2000) but may also hinder risk perception and acceptance of new environmental practices (De Dominicis et al., 2015). However, we found no (moderate or strong) correlation between place attachment and the other EDS dimensions, except for a sense of control. Future (qualitative) research is needed to further explore the role of sense of place, and other possible moderating factors including coping mechanisms (Wullenkord and Reese, 2021), and the influence of environmental awareness and education, in the environmental distress response. The observation that youth with occupations related to climate and nature (environmental organisations) reported a higher sense of control to improve the environment was indicative of convergent validity, a subtype of construct validity (Larsen et al., 2025).

### Strengths and Limitations

Our research is a first exploration of environmental distress and solastalgia, in Western young adults. Our Dutch version of the Environmental Distress Scale (EDS) appeared well applicable at population level. We explored four moderating factors in the environmental distress response, of which personality and ‘trust’ seemed to have little effect on the four different dimensions of the scale. Further exploration of ‘place attachment’ and ‘sense of control’ is needed. Our study was also influenced by several weaknesses of which the five most relevant are now listed below.

First, the original EDS has been applied and validated in the context of intensive mining in the Upper Hunter Valley of Australia, while we studied community level impact at a national (population) level. Our Dutch population was also not experiencing such clear source of environmental degradation as in the case of the intensive mining. In our research, people were asked about more chronic and possibly accumulating environmental stressors. Translations of the original ‘solastalgia’ and ‘place attachment’ dimensions to our Dutch population might have caused differences in interpretation and applicability of the original items, especially because the original EDS was validated in a relatively older population compared to our young adults. Second, our study was not preceded by a qualitative stage, such as done in the original research by Connor et al. (2004). Trying to understand perceptions and experiences, such qualitative study would have added value to our design of the Dutch EDS and the interpretation of findings.

Furthermore, our study was limited to examining four moderating factors, place attachment, sense of control, trust, and personality. However, the environmental distress response is influenced by a complex array of factors that interact and accumulate over time. Our findings indicated no significant impact of the moderating factors on individual EDS items. Nevertheless, we did not investigate their influence on the total EDS score. Given the relatively low Cronbach’s alpha for the ‘sense of control’ factor, future research should focus on refining this aspect of the questionnaire.

Additionally, assessing environmental exposure poses challenges, as people’s lives are dispersed across various activity locations and influenced by their ‘residential mobility’ over time (Helbich, 2018b). Fifth, the EDS questionnaire includes limited questions on coping mechanisms, despite their significant role in shaping the environmental stress response. Future research should enhance our understanding of the interplay between coping mechanisms and environmental distress, to design effective interventions and support systems that foster coping and adaptive engagement with environmental issues.

## Conclusion

Although a significant part of our surveyed young adults experienced environmental stress and solastalgia, an even larger number appeared somewhat ‘emotionally indifferent’ towards the state of their natural surroundings. To safeguard mental well-being and empowerment of the former group, as well as implement strategies to elevate environmental awareness and foster active engagement in the latter, more information on fundamental motivations and coping mechanisms is needed. Such knowledge aids in understanding the complexity of mental well-being in today’s young people, to provide tailored support to increase mental resilience, increase environmental awareness and environmental engagement, and empowerment of this ‘generation of hopes and dreams’.

## Supplementary Information

Below is the link to the electronic supplementary material.

Supplementary file1 (DOCX 185 KB)

Supplementary file2 (PDF 389 KB)

Supplementary file3 (PDF 319 KB)

Supplementary file4 (DOCX 23 KB)

Supplementary file5 (DOCX 88 KB)

## Data Availability

The datasets during and/or analysed during the current study available from the corresponding author on reasonable request.

## Footnotes

1 https://www.moa.nl/gouden-standaard-expertise-center.html

2 https://www.cbs.nl/nl-nl/visualisaties/dashboard-bevolking

3 https://www.flycatcher.eu/en/Home/OverOns

4 https://www.cbs.nl/nl-nl/visualisaties/dashboard-bevolking
