# Supplementary material for: Environmental Distress Among Dutch Young Adults: Worried Minds or Indifferent Hearts?
Source: Ecohealth. 2025 May 27;22(2):279–95. doi: 10.1007/s10393-025-01717-x (PMC12259751; doi:10.1007/s10393-025-01717-x)
Supplement: Supplementary file 1 — Supplementary file1 (DOCX 185 KB) [file 10393_2025_1717_MOESM1_ESM.docx]

| Part | Instrument | Items | Scale^1^ | Note | α |
| --- | --- | --- | --- | --- | --- |
| A. | Demographics |  |  | See original questionnaire, Supplementary file 1. |  |
|  | (Mental)health | 9 | 0-100 | Adjustable slider bars |  |
| B | Frequency of  environmental  stressors | 10 | 1-5 | Air pollution, noise, odour, vibrations, pollution of land and soil, pollution of water, disappearance of nature, heat, drought, and flooding were selected and adapted from frequency of hazard events scale.^2,3^ | 0.87 |
| C | Threat to self-and/or family members | 10 | 1-6 | When pp. scored ≥1 on a stressor in their home environment (D) this item was shown. Score 6 was defined as ‘unsure.’ ^2^ | 0.94 |
| D | Felt impact | 20 | 1-5 | Whether stressors impacted pp. daily functioning, either physical, psychological, cognitive, and social functioning, including depression, anxiety, stress, loss of concentration, worrying, sleeping disturbance, daily functioning (at home, in hobby/sports/social activities, and work/study), experience of impaired physical health related to the stressor(s), and one on expected future effect on ‘living environment satisfactory.’  Includes eight items from original ‘felt impact’ component of Higginbotham (2006) instrument (items 52-53, 55-56, 62, 64, 67, 69).  For item 10,15 and 17, there was an extra score 6 , defined as ‘Not applicable. ^‘2^ | 0.91 |
| E | Solastalgia | 9 | 1-6 | Translated from original list of Higginbotham ea. (2006), with the only difference that item 6 is changed from ‘farming lifestyle’ to ‘my lifestyle,’ to suit the broader public. Score 6 was defined as ‘Not applicable.’ ^2^ | 0.89 |
| F | Place attachment* | 9 | 1-5 | Translated from original list of Higginbotham ea. (2006). Item seven of the original scale was not included, and item 10 reverse scored, following advice by Higginbotham ea. (2006). ^2^ | 0.790 |
| G | Sense of control*  Extra item | 4  1 | 1-5  1-5 | Adjusted by the author, instead of the original 14 item ‘Y/N’ category ‘activities’ from Higginbotham (2006)^2.^ Includes one’s sense of control and influence, e.g. ‘I can do something myself to make my living environment healthier’ or ‘My way of life causes damage to the environment where I live.’  Higginbotham’s item on ‘economic loss’ (item 51 felt impact) was added here (extra item, not counting for sense of control score). | 0.65 |
| H | Trust* | 9 | 1-6 | Adjusted from the 8-item category of Higginbotham (2006)^2^, to suit the Dutch context. Score 6 was defined as ‘unsure.’ | 0.85 |
| I | Personality* | 10 | 1-6 | BFI-10 |  |

**Supplementary file 1**

**Psychometric details of questionnaire components**

**Table S1 Measurement instruments**

*Note*. **NL**= Netherlands. **pp**.= participants. *=moderating factors.

^1^ Scale from strongly agree (5) to strongly disagree (1), or never (1) to nearly always (5), see Appendix I. ^2^ Items were derived from [Higginbotham](https://www.researchgate.net/publication/227239947_Validation_of_an_Environmental_Distress_Scale) et al. (2006) and the translation from English to Dutch is described in section 2.3.3 below. ^3^ Items were selected for their applicability in NL by 'expert opinion’ at the municipality department of Environmental Health (GGD Groningen, NL) and their local prevalence according to the ‘[Atlas Leefomgeving](https://www.atlasleefomgeving.nl)’ and ‘Healthy living environment’ of the Dutch National institute of Public Health and the Environment (RIVM, 2023).
