# Supplementary material for: Environmental Distress Among Dutch Young Adults: Worried Minds or Indifferent Hearts?
Source: Ecohealth. 2025 May 27;22(2):279–95. doi: 10.1007/s10393-025-01717-x (PMC12259751; doi:10.1007/s10393-025-01717-x)
Supplement: Supplementary file 2 — Supplementary file2 (PDF 389 KB) [file 10393_2025_1717_MOESM2_ESM.pdf]

Supplementary file 2
Original questionnaire, Dutch

Klimaat en een gezonde leefomgeving: uw belevingen

<u>Introductie</u>

Beste heer/mevrouw,

Welkom bij de vragenlijst over klimaat en een gezonde leefomgeving.

Hiermee informeren we u over uw deelname aan dit wetenschappelijk onderzoek. Meedoen is vrijwillig en u kunt op ieder moment stoppen. Wilt u deze informatie goed doorlezen en daarna beslissen of u wilt meedoen?

Als u wilt meedoen, kunt u de vraag onder deze informatie met "ik wil meedoen aan het onderzoek" beantwoorden.
Wilt u liever toch niet meedoen, dan klikt u op "ik wil niet meedoen aan het onderzoek".

Wat is het doel van het onderzoek?
Dit onderzoek is opgezet om meer te leren over de gevoelens en gedachten die u heeft over veranderingen in uw leefomgeving en klimaatverandering.

De vragenlijst bestaat uit verschillende delen. We stellen u eerst enkele korte vragen over uw algemene gezondheid en geluksgevoel. Daarna vragen we u naar uw belevingen over uw leefomgeving. Met "leefomgeving" bedoelen we de directe omgeving waar u woont en leeft. Het gaat om uw wijk en de rand van uw wijk. De leefomgeving bestaat uit alles in uw omgeving, zoals de huizen, de lucht, uw tuin, een speeltuin of een park.

Met de tijd kan uw leefomgeving veranderen. Als de leefomgeving prettig is en uitnodigt tot gezond en sociaal gedrag, voelen mensen zich beter. Denk aan een mooi uitzicht of een fijn bos in de buurt waar u kunt wandelen en tot rust komt na een drukke dag. Soms is het minder prettig, bij voorbeeld bij overlast of hinder. Graag willen we meer leren over uw leefomgeving en de gevolgen voor uw gezondheid en geluk.

Het laatste deel van de vragenlijst gaat over klimaatverandering. De gemiddelde temperatuur op de wereld stijgt. De aarde warmt op. We noemen dit klimaatverandering. Wij zouden graag meer willen leren over uw belevingen over het klimaat.

Hoe verloopt het onderzoek?
Wij vragen u de vragenlijst volledig in te vullen. Er is geen "goed of fout" antwoord, het gaat erom hoe u de zaken beleeft. U blijft anoniem, ook al vragen we enkele algemene gegevens, zoals uw leeftijd en een deel van de postcode, zodat we bijvoorbeeld mensen die in verschillende provincies wonen, kunnen vergelijken. We zullen deze data zorgvuldig bewaren.

Hoelang duurt het onderzoek?
Doet u mee met het onderzoek? Dan duurt dat in totaal ongeveer 15 minuten.

Wie kan meedoen?
U kunt meedoen aan het vragenlijst onderzoek als u 16 jaar of ouder bent en in Nederland woont. De vragenlijst is opgesteld in het Nederlands.

Welke afspraken maken we met u?

We willen graag dat het onderzoek goed verloopt. Wij vragen u daarom om de tijd te nemen om alle vragen te beantwoorden.

Stel uw vragen

U kunt met uw vragen over dit onderzoek ook altijd terecht bij de onderzoeker van dit onderzoek XXXX

Ook kunt u over dit onderzoek praten met uw partner, familie of vrienden.

In deze informatiebrief [link] vindt u aanvullende informatie over het onderzoek.

Toestemming

Lees onderstaande punten goed door. Indien u aangeeft dat u wilt meedoen aan het onderzoek, gaat u akkoord met deze punten:

- Ik heb de informatiebrief gelezen. Ook kon ik vragen stellen. Mijn vragen zijn goed genoeg beantwoord. Ik had genoeg tijd om te beslissen over deelname.
- Ik weet dat meedoen vrijwillig is. Ook weet ik dat ik op ieder moment kan beslissen om te stoppen. Ik hoef dan niet uit te leggen waarom ik wil stoppen.
- Ik geef de onderzoekers toestemming om mijn gegevens te verzamelen en te gebruiken.
- Ik geef de onderzoekers toestemming om de anonieme gegevens te publiceren bij de artikelen die worden geschreven over dit onderzoek.
- Ik geef de onderzoekers toestemming om de anonieme gegevens, als de onderzoeker klaar is met het promotie onderzoek, voor andere onderzoekers online beschikbaar te stellen.

o ik wil meedoen aan het onderzoek
o ik wil niet meedoen aan het onderzoek [einde vragenlijst]

Algemene gegevens

We vragen u nu eerst een aantal algemene gegevens, zoals uw leeftijd, geslacht en beroep. Dit algemene deel bestaat uit 13 korte vragen.

Wilt u de antwoorden zo goed mogelijk invullen? Zo kunnen we uw antwoorden goed vergelijken met andere mensen die de vragenlijst invullen. Alle antwoorden die u geeft, worden anoniem verwerkt.

Vraag 1 Wat zijn de 4 cijfers van uw postcode?

____

ik woon niet in Nederland [einde vragenlijst]

Vraag 2 Ik identificeer mij als:

- ○ vrouw (1)
- ○ man (2)
- ○ overig (4)
- ○ wil ik niet zeggen (3)

Vraag 3 Wat is uw leeftijd?

____ jaar [indien 15 jaar of jonger of 36 jaar of ouder, einde vragenlijst]

Vraag 4a Wat is de hoogste opleiding die u heeft <u>voltooid</u>? (m.u.v. basisonderwijs wil dit zeggen dat u een diploma voor deze opleiding behaald heeft)

- o geen of basisonderwijs
- o LBO / VMBO (kader- of beroepsgericht) / MBO 1 / VBO
- o MAVO / HAVO of VWO (overgegaan naar 4e klas) / VMBO (theoretisch of gemengd) / (M)ULO
- o MBO 2, 3, 4 of MBO vóór 1998
- o HAVO of VWO (met diploma afgerond) / HBS / MMS
- o HBO propedeuse
- o HBO bachelor (of HBO vóór 2002)
- o HBO master
- o universitair propedeuse
- o universitair bachelor / kandidaats
- o universitair master / doctoraal / postdoctoraal

Vraag 4b Volgt u op dit moment een opleiding aan een school of universiteit?

- o nee
- o ja, basisonderwijs
- o ja, mavo, vmbo
- o ja, havo, atheneum, gymnasium of vwo
- o ja, LBO
- o ja, MBO
- o ja, HBO
- o ja, universiteit

Vraag 5 Wat is uw burgerlijke staat?

- ○ getrouwd (1)
- ○ geregistreerd partnerschap (2)
- ○ samenwonend (3)
- ○ lange afstandsrelatie (4)
- ○ alleenstaand (5)
- ○ gescheiden / uit elkaar (6)
- ○ weduwe/weduwnaar (7)
- ○ anders, namelijk: …

Vraag 6 In wat voor huis woont u?

- ○ sociale huurwoning (1)
- ○ particuliere huurwoning (2)
- ○ koopwoning (hypotheek) (3)
- ○ koopwoning (eigendom / hypotheek is afbetaald) (4)
- ○ onderhuur (5)
- ○ instelling, zoals bejaardenwoning (7)
- ○ detentie / gevangenis (10)
- ○ anders, namelijk (8) ____
- ○ weet ik niet

Vraag 7 **Met hoeveel personen (inclusief uzelf) woont u in uw huishouden?**
… personen van 18 jaar of ouder
… personen jonger dan 18 jaar

Vraag 8 Woont u in stedelijk gebied of daarbuiten?

- ◯ stedelijk gebied (1)
- ◯ daarbuiten (4)

Vraag 9 Hoe lang woont u al in uw uw wijk?

- ◯ korter dan 1 jaar (1)
- ◯ tussen 1 en 5 jaar (2)
- ◯ tussen 5 en 10 jaar (3)
- ◯ 10 jaar of langer (4)

Vraag 10 Wat is uw werksituatie?

- ◯ fulltime werk (meer dan 32 uur per week) (1)
- ◯ parttime werk (minder dan 32 uur per week) (2)
- ◯ bijbaan (werk naast school of studie) (3)
- ◯ vrijwilligerswerk als voornaamste bezigheid (4)
- ◯ werkloos / werkzoekend (5) [vraag 11 overslaan]
- ◯ ik werk niet en ook niet op zoek (6) [vraag 11 overslaan]
- ◯ gepensioneerd/AOW (7) [vraag 11 overslaan]
- ◯ afgekeurd (8) [vraag 11 overslaan]
- ◯ anders, namelijk (9) ____ [vraag 11 overslaan]

Vraag 11 Heeft u een beroep dat sterk verbonden is met de natuur en/of klimaat?

- ◯ ja, ik ben boswachter (1)
- ◯ ja, ik werk in de agrarische sector (boer) (2)
- ◯ ja, ik werk in natuurbeheer (3)
- ◯ ja, ik werk als klimaatwetenschapper (4)
- ◯ ja, ik werk als (5) ____
- ◯ nee (6)

Vraag 12 Wat is het bruto jaarinkomen van uw huishouden? (dit is het bruto jaarsalaris van alle leden van het huishouden, inclusief vakantiegeld en 13e maand)

- o minimum (minder dan € 14.300)
- o beneden modaal (tussen € 14.300 en € 37.000)
- o modaal (tussen € 37.000 en € 44.100)
- o tussen 1 en 2 keer modaal (tussen € 44.100 en € 74.000)
- o 2 keer modaal of meer (€ 74.000 of meer)
- o weet niet/wil niet zeggen

Vraag 13 Bent u actief in een klimaatorganisatie of ander organisatie nauw betrokken bij klimaatverandering en daarmee verbonden problemen?

X ja
X nee

<u>Deel 1: Uw gezondheid en geluksgevoel</u>

We beginnen met vier algemene vragen over uw persoonlijkheid, gezondheid en geluksgevoel.

Vraag 1 Uw **<u>Persoonlijkheid.</u>**

Hoe iemand omgaat met veranderingen en problemen, hangt erg af van iemands persoonlijkheid. We zouden daarom graag iets meer weten van uw persoonlijkheid.

Ik zie mezelf als iemand die...

	helemaal eens	eens	niet eens, niet oneens.	oneens	helemaal oneens	weet ik niet
1. gesloten is. (1)	○	○	○	○	○	○
2. goed van vertrouwen is. (2)	○	○	○	○	○	○
3. degelijk werk levert (bijv. op het werk, op school / studie en/of in het huishouden). (3)	○	○	○	○	○	○
4. ontspannen en stressbestendig is. (4)	○	○	○	○	○	○
5. een groot voorstellingsvermogen / grote fantasie heeft. (5)	○	○	○	○	○	○
6. sociaal en prettig in de omgang is. (6)	○	○	○	○	○	○
7. neigt naar fouten bij anderen zoeken. (7)	○	○	○	○	○	○
8. neigt lui te zijn. (8)	○	○	○	○	○	○
9. snel nerveus wordt. (9)	○	○	○	○	○	○
10. weinig kunstzinnige interesses heeft. (10)	○	○	○	○	○	○

Vraag 2 **<u>Gevoelens</u>**

We zouden graag willen weten hoe u zich het afgelopen jaar over het algemeen voelde.

<u>Geef een cijfer van 0 tot 100 door de balk te verschuiven.</u>
Een cijfer 0 betekent “helemaal niet”.

Een cijfer 100 betekent “heel erg”.

	0 10 20 30 40 50 60 70 80 90 100
Hoe **gelukkig** voelde u zich in het afgelopen jaar? ()	
Hoe **somber** voelde u zich in het afgelopen jaar? ()	
Hoe **angstig** voelde u zich in het afgelopen jaar? ()	
Hoe **eenzaam** voelde u zich in het afgelopen jaar? ()	
Hoe **gespannen** voelde u zich in het afgelopen jaar? ()	

Vraag 3 **<u>Uw gezondheid.</u>**

Hoe gezond vindt u dat u bent? En hoe gezond is uw leefomgeving, uw wijk, voor u?

<u>Geef een cijfer van 0 tot 100 door de balk te verschuiven.</u>
Een cijfer 0 betekent “heel slecht”
Een cijfer 100 betekent “ heel erg goed”.

Met uw “leefomgeving” bedoelen we de plek waar u woont en leeft. Het gaat om uw wijk en de rand van uw wijk.
Het gaat om zaken als de huizen, de lucht, uw tuin, een speeltuin of een park.

	0 10 20 30 40 50 60 70 80 90 100
Mijn eigen gezondheid, nu. ()	
De gezondheid van mijn leefomgeving, nu. ()	

Vraag 4 **<u>Uw gezondheid over 10 jaar.</u>**
Hoe verwacht u dat uw gezondheid over 10 jaar is? En de gezondheid van uw leefomgeving, uw wijk?

<u>Geef een cijfer van 0 tot 100 door de balk te verschuiven.</u>
Een cijfer 0 betekent “heel slecht”.
Een cijfer 100 betekent “ heel erg goed””.

Met uw “leefomgeving” bedoelen we de plek waar u woont en leeft. Het gaat om uw wijk en de rand van uw wijk. Het gaat om zaken als de huizen, de lucht, uw tuin, een speeltuin of een park.

	0 10 20 30 40 50 60 70 80 90 100
Mijn eigen gezondheid, over 10 jaar. ()	
De gezondheid van mijn leefomgeving, over 10 jaar. ()	

Dit is het einde van deel 1.
Heeft u opmerkingen over deel 1? Deze kunt u hieronder invullen.

____

Deel 2 Uw leefomgeving

De volgende 5 vragen gaan over uw leefomgeving.

Met "leefomgeving" bedoelen we de directe omgeving waar u woont en leeft. Het gaat om uw wijk en de rand van uw wijk. De leefomgeving bestaat uit alles in uw omgeving, zoals de huizen, de lucht, uw tuin, een speeltuin of een park.

Met de tijd kan uw leefomgeving veranderen. Als de leefomgeving prettig is en uitnodigt tot gezond en sociaal gedrag, voelen mensen zich beter. Soms is het minder prettig, bijvoorbeeld bij overlast of hinder. Graag willen we meer leren over uw leefomgeving en de gevolgen voor uw gezondheid en geluk.

Een emotionele band met uw leefomgeving.
Een gezonde leefomgeving is belangrijk. We brengen er veel tijd door. Mensen wonen vaak hun hele leven op dezelfde plek. Dit kan ervoor zorgen dat mensen een emotionele band voelen met hun leefomgeving.

Vraag 5 Hoe is dit voor u: voelt u een emotionele band met uw leefomgeving?

	helemaal eens	eens	niet eens en niet oneens (neutraal)	oneens	helemaal oneens
1. Ik ben trots op het erfgoed van deze plek. (info-button: Erfgoed is de nalatenschap, erfenis, alles wat er al was bij generaties die voor u leefden)	○	○	○	○	○
2. Ik zou op deze plek blijven wonen, zelfs	○	○	○	○	○

als ik de kans zou krijgen om te vertrekken.					
3. Mijn gevoel van wie ik ben, is verbonden aan de omgeving waarin ik woon.	○	○	○	○	○
4. Ik krijg troost of rust (in mijn hoofd) van deze plek.	○	○	○	○	○
5. Ik voel dat ik iedere rots, hoekje of gaatje hier ken.	○	○	○	○	○
6. Ik voel me diep verbonden met deze plek.	○	○	○	○	○
7. Ik zou liever ergens anders wonen; dit is niet de plek voor mij.	○	○	○	○	○
8. Ik voel een verantwoordelijkheid voor de mensen van deze plek.					
9. Ik voel dat ik de plicht heb om het land te behouden voor toekomstige generaties.	○	○	○	○	○
10. Door de veranderingen op deze plek, zou ik vertrekken als ik kon.					

U kunt aangeven in hoeverre u het eens of oneens bent met de volgende stellingen.

<u>Veranderingen in uw leefomgeving.</u>

In de loop van de tijd kan uw leefomgeving veranderen. Dit kan leuk zijn, maar sommige zaken kunnen ook tot hinder of overlast zorgen.

Als u veranderingen opmerking in uw omgeving, hoe is dat voor u?

Met uw “leefomgeving” bedoelen we de plek waar u woont en leeft. Het gaat om uw wijk en de rand van uw wijk. Het gaat om zaken als de huizen, de lucht, uw tuin, een speeltuin of een park.

Vraag 6 Wilt u aangeven in hoeverre u het eens of oneens bent met de stellingen?

	helemaal eens	eens	niet eens, niet oneens (neutraal)	oneens	helemaal oneens	niet van toepassing
1. Ik voel mij minder thuis door ongewenste verandering(en).	○	○	○	○	○	○
2. Ik ben verdrietig dat vertrouwde onderdelen van deze plek verdwijnen (bijvoorbeeld dieren, planten, oriëntatiepunten, open ruimte).	○	○	○	○	○	○
3. Ik ben bezorgd dat onderdelen van dit gebied die ik waardeer, verloren gaan.	○	○	○	○	○	○
4. Ik mis het vredige gevoel waar ik ooit van genoot als ik op deze plek was.	○	○	○	○	○	○
5. Ik ben van streek over hoe deze omgeving er nu uitziet.	○	○	○	○	○	○
6. Mijn manier van leven wordt bedreigd door verandering(en) in	○	○	○	○	○	○

mijn lokale omgeving.						
7. Unieke onderdelen van de natuur die deze plek bijzonder maakten, gaan voor altijd verloren.						
8. Ik ben bedroefd door ongewenste verandering(en) die ik in mijn landschap zie.	○	○	○	○	○	○
9. Ik voel me machteloos om ongewenste verandering(en) op deze plek tegen te houden.	○	○	○	○	○	○

Hinder in uw omgeving.

Vraag 7 Sommige veranderingen in uw omgeving kunnen zorgen voor hinder of overlast. Kunt u van onderstaande aspecten voor <u>afgelopen jaar</u> aangeven of u ze heeft meegemaakt?

Met uw "leefomgeving" bedoelen we de plek waar u woont en leeft. Het gaat om uw wijk en de rand

van uw wijk. Het gaat om zaken als de huizen, de lucht, uw tuin, een speeltuin of een park.

	nooit	zelden	soms	vaak	(bijna) altijd
1. luchtvervuiling (bijv. stof, rook, smog) (1)	○	○	○	○	○
2. geluid (bijv. door industrie en/of verkeer) (2)	○	○	○	○	○
3. stank (bijv. door industrie en/of verkeer) (3)	○	○	○	○	○
4. trillingen (bijv. door industrie, verkeer) (4)	○	○	○	○	○
5. vervuiling van land en bodem (bijv. door chemische stoffen, bestrijdingsmiddelen) (5)	○	○	○	○	○
6. vervuiling van het water in sloten en rivieren (6)	○	○	○	○	○
7. verdwijnen van natuur (bijv. door bouw van huizen of industrie) (7)	○	○	○	○	○
8. hitte (8)	○	○	○	○	○
9. schade aan de omgeving door droogte (9)	○	○	○	○	○
10. wateroverlast door regen of overstroming (10)	○	○	○	○	○

[Indien overal nooit is beantwoord op vraag 7, dan door naar vraag 10]

<u>Invloed op gezondheid en geluksgevoel.</u>

Vraag 8 In de vorige vraag heeft u aangegeven afgelopen jaar een of meerdere zaken mee te hebben gemaakt in uw leefomgeving. Voelen deze zaken als een “dreiging” voor u of die van uw familieleden?
Met een “dreiging” bedoelen we dat het voor u voelt als (mogelijk) slecht voor uw geluksgevoel en/of gezondheid.
[alleen die opties waar bij vraag 7 zelden, soms, vaak of bijna altijd zijn gekozen, worden hier weergegeven]

	geen dreiging	lage dreiging	matige dreiging	sterke dreiging	heel sterke dreiging	ik weet het niet
1. luchtvervuiling (bijv. stof, rook, smog) (1)	○	○	○	○	○	○
2. geluid (bijv. door industrie en/of verkeer) (2)	○	○	○	○	○	○
3. stank (bijv. door industrie en/of verkeer) (3)	○	○	○	○	○	○
4. trillingen (bijv. door industrie, verkeer) (4)	○	○	○	○	○	○
5. vervuiling van land en bodem (bijv. door chemische stoffen, bestrijdingsmiddelen) (5)	○	○	○	○	○	○
6. vervuiling van het water in sloten en rivieren (6)	○	○	○	○	○	○
7. verdwijnen van natuur (bijv. door bouw van huizen of industrie) (7)	○	○	○	○	○	○
8. hitte (8)	○	○	○	○	○	○
9. schade aan de omgeving door droogte (9)	○	○	○	○	○	○
10. wateroverlast door regen of overstroming (10)	○	○	○	○	○	○

Gevolgen voor het dagelijks leven.

Vraag 9 U heeft aangegeven dat uw leefomgeving verandert, bijvoorbeeld door toename van geluid of luchtvervuiling.
Hebben deze veranderingen invloed op uw geluksgevoel en dagelijks functioneren? Het gaat hier weer om afgelopen jaar. Denk dus aan de periode van vandaag tot een jaar geleden.
Door de verandering(en) in mijn leefomgeving..

	helemaal eens	eens	niet eens, niet oneens (neutraal)	oneens	helemaal oneens	weet niet / n.v.t. (alleen voor stelling 10, 15 en 17)
1. kan ik minder van het leven genieten. (1)	○	○	○	○	○	○
2. word ik vooral blij. (2)	○	○	○	○	○	○
3. word ik somber. (3)	○	○	○	○	○	○
4. ben ik boos. (4)	○	○	○	○	○	○
5. ben ik angstig. (5)	○	○	○	○	○	○
6. heb ik spanning, stress. (6)	○	○	○	○	○	○
7. kan me mij minder goed concentreren. (7)	○	○	○	○	○	○
8. pieker ik veel. (8)						

9. kan ik niet goed slapen. (9)						
10. kan ik mijn werk niet goed doen / kan ik minder goed studeren / leren. (10)						
11. kan ik thuis minder goed functioneren. (11)						
12. kan ik juist beter functioneren. (12)						
13. heb ik gezondheidsklachten. (13)						
14. voel ik me belemmerd in mijn hobby’s, sport, uitgaan. (14)						
15. denk ik dat mensen in mijn leefomgeving / wijk er ziek van worden. (15)						
16. denk ik dat ik daar zelf ziek van kan worden. (16)						
17. is mijn huis minder waard. (17)						
18. zijn er spanningen en/of ruzie in de wijk. (18)						
19. heb ik zorgen over de toekomst. (19)						
20. wordt het in mijn wijk komende jaren alleen maar fijner om te leven. (20)						

Uw eigen invloed op de leefomgeving.

Vraag 10 Vindt u dat u zelf iets kunt doen om de gezondheid van uw leefomgeving beter te maken?

Wilt u aangeven in hoeverre u het eens of oneens bent met de volgende vijf stellingen?

Met uw “leefomgeving” bedoelen we de plek waar u woont en leeft. Het gaat om uw wijk en de rand

van uw wijk.
Het gaat om zaken als de huizen, de lucht, uw tuin, een speeltuin of een park.

	helemaal eens	eens	niet eens, niet oneens (neutraal)	oneens	helemaal oneens
1. Ik kan zelf iets doen om mijn leefomgeving gezonder te maken. (1)	○	○	○	○	○
2. Mijn manier van leven veroorzaakt schade aan de leefomgeving waar ik woon. (2)	○	○	○	○	○
3. Ik heb invloed op beslissingen die gaan over mijn leefomgeving. (3)	○	○	○	○	○
4. Ik ben zelf bezig met beschermen en gezond houden van mijn leefomgeving. (4)	○	○	○	○	○
5. De economie en/of werkgelegenheid (banen) zijn belangrijker voor mij dan het hebben van een gezonde leefomgeving. (5)	○	○	○	○	○

Dit is het einde van deel 2.
Heeft u opmerkingen over deel 2? Deze kunt u hieronder invullen.

____

Klik op Volgende om verder te gaan met deel 3.
Deel 3 Klimaatverandering Vraag 11-16. ***Not included in the results of this paper.***

Vertrouwen in instanties

Vraag 17 **Wilt u aangeven of u vertrouwen heeft in de volgende personen en instanties, als het gaat om de aanpak van omgevingsbedreigingen waaronder klimaatverandering?**

	helemaal geen vertrouwen (1)	weinig vertrouwen (2)	beetje vertrouwen (3)	vertrouwen (4)	heel veel vertrouwen (5)	ik weet het niet/geen mening (6)
1. uw gemeente (1)	○	○	○	○	○	○
2. uw huisarts (2)	○	○	○	○	○	○
3. de Nederlandse overheid, regering (3)	○	○	○	○	○	○
4. artsen en verpleegkundigen (4)	○	○	○	○	○	○
5. de Nederlandse Publieke Gezondheidszorg (RIVM, GGD's) (5)	○	○	○	○	○	○
6.internationale organisaties, zoals Intergovernmental Panel on Climate Change (IPCC), World Health Organization (WHO). (6)	○	○	○	○	○	○
7. industrie (7)	○	○	○	○	○	○
8 klimaat-organisaties, zoals Milieudefensie, Extinction Rebellion (8)	○	○	○	○	○	○
9. Europese beleidsmakers, zoals de Europese Unie (9)	○	○	○	○	○	○

Duurzaam leven

Nu volgen nog 2 open vragen waar u zelf de antwoorden kunt opschrijven.

Not included in the results of this paper.

Dit is het einde van de vragenlijst.

Hartelijk bedankt voor uw deelname aan deze vragenlijst.

Heeft u opmerkingen over dit deel van de vragenlijst? Hieronder is ruimte voor uw opmerkingen.

____

____

Klik op Volgende om uw antwoorden te versturen.
