# Supplementary material for: Environmental Distress Among Dutch Young Adults: Worried Minds or Indifferent Hearts?
Source: Ecohealth. 2025 May 27;22(2):279–95. doi: 10.1007/s10393-025-01717-x (PMC12259751; doi:10.1007/s10393-025-01717-x)
Supplement: Supplementary file 3 — Supplementary file3 (PDF 319 KB) [file 10393_2025_1717_MOESM3_ESM.pdf]

Supplementary file 3

Translated questionnaire, English

Climate and a Healthy Living Environment: Your Experiences

Translated from original Dutch questionnaire: ‘Klimaat en een gezonde leefomgeving: uw belevingen’

Introduction

Dear Sir/Madam,

Welcome to the survey about climate change and a healthy living environment.

This document provides information about your participation in this scientific study. Participation is voluntary, and you may stop at any time. Please read this information carefully and then decide if you want to participate.

If you wish to participate, please answer the question below with “I want to participate in the study.” If you prefer not to participate, please select “I do not want to participate in the study.”

What is the purpose of the research?
This research aims to learn more about your feelings and thoughts regarding changes in your living environment and climate change. The questionnaire consists of several parts. First, we ask you a few general questions about your health and happiness. Then, we will inquire about your experiences regarding your living environment. By "living environment," we mean the immediate area where you live, including your neighborhood and its outskirts. It encompasses everything around you, such as houses, the air, your garden, a playground, or a park.

Over time, your living environment can change. When the environment is pleasant and encourages healthy and social behavior, people feel better. For example, a nice view or a peaceful forest nearby where you can walk and relax after a busy day. Sometimes, the environment can be less pleasant, such as in cases of noise or pollution. We would like to learn more about your living environment and its impact on your health and happiness.

The last part of the survey concerns climate change. The Earth's average temperature is rising, and the planet is warming. This is called climate change. We would like to learn more about your perceptions of the climate.

How does the study work?
We ask you to complete the entire questionnaire. There are no "right or wrong" answers—this is about your personal experience. You will remain anonymous, even though we will ask for some general information, such as your age and part of your postal code, so we can compare responses from people living in different provinces, for example. We will handle this data with care.

How long does the research take?
If you choose to participate, the questionnaire will take approximately 15 minutes to complete.

Who can participate?
You can participate if you are 16 years or older and live in the Netherlands. The survey is available in Dutch.

What are the terms and conditions?
We want the study to run smoothly, so we kindly ask you to take your time in answering all the questions.

Ask Your Questions
If you have questions about the study, you can always contact the researcher, XXXX.

You may also discuss the study with your partner, family, or friends.

Additional information about the study can be found in this information sheet [link].

Consent
Please read the points below carefully. By selecting that you want to participate, you agree to these terms:

- I have read the information sheet and had the opportunity to ask questions. My questions have been answered satisfactorily. I had enough time to decide whether to participate.
- I understand that participation is voluntary. I also understand that I can stop at any time without providing an explanation.
- I give the researchers permission to collect and use my data.
- I give the researchers permission to publish anonymous data in articles related to this study.
- I give the researchers permission to make the anonymous data available online to other researchers once the study is completed.
    - o I want to participate in the study.
    - o I do not want to participate in the study. [end of the questionnaire]

General Information

We now ask you some general questions about your age, gender, and occupation. This section contains 13 short questions.

Please answer the questions as accurately as possible, so we can compare your responses with those of other participants. All your answers will be processed anonymously.

Question 1 What are the 4 digits of your postal code?

I do not live in the Netherlands [end of the questionnaire]

Question 2 I identify as:
o woman (1)
o man (2)
o other (4)
o prefer not to say (3)

Question 3 What is your age?
____ years [if 15 years or younger or 36 years or older, end of the questionnaire]

Question 4a What is the highest level of education you have completed? (excluding primary school, meaning you have obtained a diploma for this level)

o none or primary school
o LBO / VMBO (framework or vocational) / MBO 1 / VBO
o MAVO / HAVO or VWO (advanced to the 4th year) / VMBO (theoretical or mixed) / (M)ULO
o MBO 2, 3, 4 or MBO before 1998
o HAVO or VWO (with diploma completed) / HBS / MMS
o HBO propaedeutic
o HBO bachelor (or HBO before 2002)
o HBO master
o university propaedeutic
o university bachelor / candidacy
o university master / doctoral / postdoctoral

Question 4b Are you currently enrolled in an educational institution (school or university)?

o no
o yes, primary school
o yes, MAVO, VMBO
o yes, HAVO, atheneum, gymnasium, or VWO
o yes, LBO
o yes, MBO
o yes, HBO
o yes, university

Question 5 What is your marital status?
o married (1)
o registered partnership (2)
o living together (3)
o long-distance relationship (4)
o single (5)
o divorced / separated (6)
o widowed (7)
o other, namely: _______________

Question 6 What type of house do you live in?
o social rental housing (1)
o private rental housing (2)
o owned home (mortgage) (3)
o owned home (mortgage paid off) (4)
o sublet (5)
o institution, such as a retirement home (7)
o detention / prison (10)
o other, namely (8) ____
o don't know

Question 7 How many people (including yourself) live in your household?
… people aged 18 or older
… people younger than 18

Question 8 Do you live in an urban area or outside of it?
o urban area (1)
o outside of it (4)

Question 9 How long have you lived in your neighborhood?
o less than 1 year (1)
o between 1 and 5 years (2)
o between 5 and 10 years (3)
o 10 years or longer (4)

Question 10 What is your employment situation?
o full-time work (more than 32 hours per week) (1)
o part-time work (less than 32 hours per week) (2)
o side job (work alongside school or study) (3)
o volunteer work as primary activity (4)
o unemployed / job seeking (5) [skip question 11]
o I am not working and not looking for work (6) [skip question 11]
o retired / receiving pension (7) [skip question 11]
o incapacitated (8) [skip question 11]
o other, namely (9) ____ [skip question 11]

Question 11 Is your occupation strongly related to nature and/or climate?
o yes, I am a forest ranger (1)
o yes, I work in the agricultural sector (farmer) (2)
o yes, I work in nature management (3)
o yes, I work as a climate scientist (4)
o yes, I work as (5) ____
o no (6)

Question 12 What is the gross annual income of your household? (This is the gross annual salary of all members of the household, including vacation pay and 13th month)

o minimum (less than €14,300)
o below average (between €14,300 and €37,000)
o average (between €37,000 and €44,100)
o between 1 and 2 times average (between €44,100 and €74,000)
o 2 times average or more (€74,000 or more)
o don’t know / prefer not to say

Question 13 Are you active in a climate organization or another organization closely involved in climate change and related issues?

X yes
X no

Part 1: Your Health and Happiness

We begin with four general questions about your personality, health, and sense of well-being.

Question 1 Your Personality.

How someone deals with changes and problems depends a lot on their personality. Therefore, we would like to know a little more about your personality.

I see myself as someone who...

	Strongly agree	Agree	Neutral	Disagree	Strongly disagree	Don't know
1. Is reserved. (1)	o	o	o	o	o	o
2. Is generally trusting. (2)	o	o	o	o	o	o
3. Does a thorough job (e.g., at work, in school/study, or in household). (3)	o	o	o	o	o	o
4. Is relaxed, handles stress well. (4)	o	o	o	o	o	o
5. Has an active imagination. (5)	o	o	o	o	o	o
6. Is outgoing, sociable. (6)	o	o	o	o	o	o
7. Tends to find faults with others. (7)	o	o	o	o	o	o
8. Tends to be lazy. (8)	o	o	o	o	o	o
9. Gets nervous easily. (9)	o	o	o	o	o	o
10. Has few artistic interests. (10)	o	o	o	o	o	o

Question 2: Feelings We would like to know how you generally felt in the past year.

Rate from 0 to 100 by sliding the bar. A 0 means “not at all,” and a 100 means “very much.”

0 10 20 30 40 50 60 70 80 90 100

- How happy did you feel in the past year? ()
- How gloomy did you feel in the past year? ()
- How anxious did you feel in the past year? ()
- How lonely did you feel in the past year? ()
- How stressed did you feel in the past year? ()

Question 3: Your Health. How healthy do you think you are? And how healthy is your living environment, your neighborhood, for you?

Rate from 0 to 100 by sliding the bar. A 0 means “very bad,” and a 100 means “very good.”

Your “living environment” refers to where you live and spend time. It includes your neighborhood and the surrounding area, such as houses, air quality, your garden, a playground, or a park.

0 10 20 30 40 50 60 70 80 90 100

- My own health, now. ()
- The health of my living environment, now. ()

Question 4: Your health in 10 years. How do you expect your health to be in 10 years? And the health of your living environment, your neighborhood?

Rate from 0 to 100 by sliding the bar. A 0 means "very bad," and a 100 means "very good."

0 10 20 30 40 50 60 70 80 90 100

- My own health, in 10 years. ()
- The health of my living environment, in 10 years. ()

This concludes Section 1.
Do you have any comments about Section 1? Please fill them in below.

Section 2: Your living environment

The following five questions are about your living environment.

By "living environment," we mean the direct surroundings where you live and spend time. It refers to your neighborhood and its borders. The living environment includes everything in your area, such as the houses, air quality, your garden, a playground, or a park. Over time, your living environment may change. If the environment is pleasant and encourages healthy and social behavior, people feel better. Sometimes, however, it may be less pleasant, for example, due to nuisance or disturbance. We would like to learn more about your living environment and its effects on your health and happiness.

Emotional connection to your living environment. A healthy living environment is important. We spend a lot of time there, and people often live in the same place their entire lives. This can create an emotional bond with the place.

Question 5: How is this for you: Do you feel an emotional connection with your living environment? You can indicate how much you agree or disagree with the following statements.

**Statement**	**Strongly agree**	**Agree**	**Neutral**	**Disagree**	**Strongly disagree**
1. I am proud of the heritage of this place.	o	o	o	o	o
2. I would continue to live in this place even if I were given the opportunity to leave.	o	o	o	o	o
3. My sense of who I am is linked to the environment where I live.	o	o	o	o	o
4. I get comfort or peace of mind from this place.	o	o	o	o	o
5. I feel like I know every rock, nook and cranny around these parts.	o	o	o	o	o
6. I feel a deep connect to this place.	o	o	o	o	o
7. I would rather live somewhere different; this is not the place for me.	o	o	o	o	o

Statement	Strongly agree	Agree	Neutral	Disagree	Strongly disagree
8. I feel a sense of responsibility to the people of this place.	o	o	o	o	o
9. I feel that I have a duty to maintain the land for future generations.	o	o	o	o	o
10. Because of the changes to this place, I would leave if I could.	o	o	o	o	o

---

Changes in your living environment. Over time, your living environment can change. Some changes may be enjoyable, but others can cause disturbances or inconveniences. If you notice changes in your surroundings, how does it affect you?

By "living environment," we mean the place where you live and spend time. It refers to your neighborhood and its borders, such as houses, air quality, your garden, a playground, or a park.

Question 6: Would you indicate how much you agree or disagree with the following statements?

Statement	Strongly agree	Agree	Neutral	Disagree	Strongly disagree	Not applicable
1. My sense of belonging to this place has been undermined by unwelcome change.	o	o	o	o	o	o
2. I am sad that familiar parts of this place are disappearing (e.g., animals, plants, landmarks, open spaces).	o	o	o	o	o	o
3. I am worried that aspects of this area that I value are being lost.	o	o	o	o	o	o
4. I miss having the sense of peace and quiet that I once enjoyed by being in this place.	o	o	o	o	o	o

5. I am upset about the way this area looks now.
6. My lifestyle is being threatened by environmental change in my local area.
7. Unique aspects of nature that made this place special are being lost forever.
8. I am saddened by unwelcome change(s) that I see in my landscape.
9. I feel powerless to stop unwanted changes to this place.

Nuisance in Your Environment

Disturbances in your environment.

Question 7: Some changes in your environment may cause nuisance or disturbance.
Can you indicate whether you experienced any of the following aspects in the past year?

By "living environment," we mean the place where you live and spend time. This refers to your neighborhood and its boundaries, including things like houses, the air, your garden, a playground, or a park.

**Aspect**	**Never**	**Rarely**	**Sometimes**	**Often**	**(Almost) Always**
1. Air pollution (e.g., dust, smoke, smog) (1)	**o**	**o**	**o**	**o**	**o**
2. Noise (e.g., from industry and/or traffic) (2)	**o**	**o**	**o**	**o**	**o**
3. Smell (e.g., from industry and/or traffic) (3)	**o**	**o**	**o**	**o**	**o**
4. Vibrations (e.g., from industry, traffic) (4)	**o**	**o**	**o**	**o**	**o**
5. Land and soil pollution (e.g., from chemicals, pesticides) (5)	**o**	**o**	**o**	**o**	**o**
6. Pollution of water in ditches and rivers (6)	**o**	**o**	**o**	**o**	**o**
7. Loss of nature (e.g., from the construction of houses or industry) (7)	**o**	**o**	**o**	**o**	**o**
8. Heat (8)	**o**	**o**	**o**	**o**	**o**
9. Damage to the environment from drought (9)	**o**	**o**	**o**	**o**	**o**
10. Water damage from rain or flooding (10)	**o**	**o**	**o**	**o**	**o**

[If “Never” is selected for all answers in Question 7, proceed to Question 10.]

Impact on health and sense of happiness.

Question 8: In the previous question, you indicated that you experienced one or more issues in your living environment last year. Do these issues feel like a "threat" to you or your family members?
By "threat," we mean that it feels (possibly) harmful to your sense of happiness and/or health.

[Only the options where "rarely," "sometimes," "often," or "almost always" were chosen in Question 7 will appear here.]

**Aspect**	**No threat**	**Low threat**	**Moderate threat**	**Strong threat**	**Very strong threat**	**I don't know**
1. Air pollution (e.g., dust, smoke, smog) (1)	**o**	**o**	**o**	**o**	**o**	**o**
2. Noise (e.g., from industry and/or traffic) (2)	**o**	**o**	**o**	**o**	**o**	**o**
3. Smell (e.g., from industry and/or traffic) (3)	**o**	**o**	**o**	**o**	**o**	**o**
4. Vibrations (e.g., from industry, traffic) (4)	**o**	**o**	**o**	**o**	**o**	**o**
5. Land and soil pollution (e.g., from chemicals, pesticides) (5)	**o**	**o**	**o**	**o**	**o**	**o**
6. Pollution of water in ditches and rivers (6)	**o**	**o**	**o**	**o**	**o**	**o**

Aspect	No threat	Low threat	Moderate threat	Strong threat	Very strong threat	I don't know
7. Loss of nature (e.g., from the construction of houses or industry) (7)	o	o	o	o	o	o
8. Heat (8)	o	o	o	o	o	o
9. Damage to the environment from drought (9)	o	o	o	o	o	o
10. Water damage from rain or flooding (10)	o	o	o	o	o	o

Consequences for daily life.

Question 9: You indicated that your living environment is changing, for example, due to increased noise or air pollution.

Do these changes affect your sense of happiness and daily functioning?
This refers to the period from today to one year ago.

Due to the changes in my living environment...

Statement	Strongly agree	Agree	Neither agree nor disagree (neutral)	Disagree	Strongly disagree	Don't know / N/A (only for statements 10, 15, and 17)
1. I’m less able to enjoy life. (1)	o	o	o	o	o	o
2. I’m experiencing joy. (2)	o	o	o	o	o	o
3. I become depressed. (3)	o	o	o	o	o	o
4. I’m angry. (4)	o	o	o	o	o	o
5. I’m anxious. (5)	o	o	o	o	o	o
6. I experience tension, stress. (6)	o	o	o	o	o	o
7. I’m less able to concentrate. (7)	o	o	o	o	o	o
8. I worry a lot. (8)	o	o	o	o	o	o
9. I cannot sleep well. (9)	o	o	o	o	o	o
10. I’m less able to perform in study and/or work. (10)	o	o	o	o	o	o
11. I’m less able to function well at home. (11)	o	o	o	o	o	o
12. I feel an improvement in my daily functioning. (12)	o	o	o	o	o	o
13. I experience health problems. (13)	o	o	o	o	o	o

Statement	Strongly agree	Agree	Neither agree nor disagree (neutral)	Disagree	Strongly disagree	Don't know / N/A (only for statements 10, 15, and 17)
14. I feel hindered in hobby/sports/social activities. (14)	o	o	o	o	o	o
15. I think people in my living environment/neighborhood are getting ill from this. (15)	o	o	o	o	o	o
16. I think I personally can become ill from this. (16)	o	o	o	o	o	o
17. Is my house worth less. (17)	o	o	o	o	o	o
18. There are tensions and/or arguments in my neighborhood. (18)	o	o	o	o	o	o
19. I have concerns about the future. (19)	o	o	o	o	o	o
20. It will only become better to live in my neighborhood in the coming years. (20)	o	o	o	o	o	o

---

Your own influence on the living environment.

Question 10: Do you think there is something you can do to improve the health of your living environment?

Please indicate how much you agree or disagree with the following five statements.

By "living environment," we mean the place where you live and spend time. This refers to your neighborhood and its boundaries, including things like houses, the air, your garden, a playground, or a park.

Statement	Strongly agree	Agree	Neither agree nor disagree (neutral)	Disagree	Strongly disagree
1. I can do something myself to make my home environment healthier. (1)	o	o	o	o	o
2. My way of living causes damage to the environment where I live. (2)	o	o	o	o	o
3. I have influence on decisions regarding my living environment. (3)	o	o	o	o	o
4. I am actively involved in protecting and improving (health of) my living environment. (4)	o	o	o	o	o
5. The economy and/or employment (jobs) are more important to me than having a healthy living environment. (5)	o	o	o	o	o

This is the end of Part 2.
Do you have any comments about Part 2? You can write them below.

Click Next to continue to Part 3.

Part 3: Climate Change
Questions 11-16.

Not included in the results of this paper.

Trust in Authorities

Question 17: Please indicate whether you have trust in the following people and organizations, regarding tackling environmental change including climate change?

	No trust at all (1)	Little trust (2)	Some trust (3)	Trust (4)	A lot of trust (5)	I don't know/No opinion (6)
1. Your municipality (1)	o	o	o	o	o	o
2. Your GP (2)	o	o	o	o	o	o
3. The Dutch government, government (3)	o	o	o	o	o	o
4. Doctors and nurses (4)	o	o	o	o	o	o
5. Dutch Public Health (RIVM, GGD's) (5)	o	o	o	o	o	o
6. International organizations, such as the Intergovernmental Panel on Climate Change (IPCC), World Health Organization (WHO) (6)	o	o	o	o	o	o
7. Industry (7)	o	o	o	o	o	o
8. Climate organizations, such as Milieudefensie, Extinction Rebellion (8)	o	o	o	o	o	o
9. European policymakers, such as the European Union (9)	o	o	o	o	o	o

---

Sustainable Living

Next, there are two open-ended questions where you can write your own answers.

Not included in the results of this paper.

This is the end of the questionnaire.

Thank you very much for participating in this survey.

Do you have any comments about this part of the questionnaire? Below is space for your comments.

Click Next to submit your answers.
