# Supplementary material for: Environmental Distress Among Dutch Young Adults: Worried Minds or Indifferent Hearts?
Source: Ecohealth. 2025 May 27;22(2):279–95. doi: 10.1007/s10393-025-01717-x (PMC12259751; doi:10.1007/s10393-025-01717-x)
Supplement: Supplementary file 4 — Supplementary file4 (DOCX 23 KB) [file 10393_2025_1717_MOESM4_ESM.docx]

**Supplementary file 4**

**Flowchart**

**Figure S1.** Flowchart showing the questionnaire response, in which 1261 respondents started the questionnaire, of which 1006 were eventually included in the data analysis.
