# Supplementary material for: Environmental Distress Among Dutch Young Adults: Worried Minds or Indifferent Hearts?
Source: Ecohealth. 2025 May 27;22(2):279–95. doi: 10.1007/s10393-025-01717-x (PMC12259751; doi:10.1007/s10393-025-01717-x)
Supplement: Supplementary file 5 — Supplementary file5 (DOCX 88 KB) [file 10393_2025_1717_MOESM5_ESM.docx]

**Supplementary file 5**

**Correlations**

**Table S2.** Spearman correlations and p-values
